# Separation and Identification of Permethylated Glycan Isomers by Reversed Phase NanoLC-NSI-MS^n^

**DOI:** 10.1074/mcp.RA120.002266

**Published:** 2021-01-26

**Authors:** Simone Kurz, M. Osman Sheikh, Shan Lu, Lance Wells, Michael Tiemeyer

**Affiliations:** 1 Complex Carbohydrate Research Center, University of Georgia, Athens, Georgia, USA; 2 Department of Medicine, University of Massachusetts Medical School, Worcester, Massachusetts, USA; 3 Department of Biochemistry and Molecular Biology, University of Georgia, Athens, Georgia, USA

**Keywords:** liquid chromatography, permethylation, glycan, reverse phase, mass spectrometry, nanoflow, nano spray ionization, ion trap, isomer, ACN, acetonitrile, AcOH, acetic acid, CFG, Consortium for Functional Glycomics, DP, degree of polymerization, EIC, extracted ion chromatogram, ITMS, ion trap mass spectrometer, LiOAc, lithium acetate, NaOH, sodium hydroxide, MeI, methyl iodide, mTEC, mouse tracheal epithelial cell, nanoLC-NSI-MS, nanoflow Liquid Chromatography-Nanospray Ionization-Mass Spectrometry, MS^n^, multidimensional ion fragmentation, RP, reverse phase

## Abstract

HPLC has been employed for decades to enhance detection sensitivity and quantification of complex analytes within biological mixtures. Among these analytes, glycans released from glycoproteins and glycolipids have been characterized as underivatized or fluorescently tagged derivatives by HPLC coupled to various detection methods. These approaches have proven extremely useful for profiling the structural diversity of glycoprotein and glycolipid glycosylation but require the availability of glycan standards and secondary orthogonal degradation strategies to validate structural assignments. A robust method for HPLC separation of glycans as their permethylated derivatives, coupled with in-line multidimensional ion fragmentation (MS^n^) to assign structural features independent of standards, would significantly enhance the depth of knowledge obtainable from biological samples. Here, we report an optimized workflow for LC-MS analysis of permethylated glycans that includes sample preparation, mobile phase optimization, and MS^n^ method development to resolve structural isomers on-the-fly. We report baseline separation and MS^n^ of isomeric N- and O-glycan structures, aided by supplementing mobile phases with Li^+^, which simplifies adduct heterogeneity and facilitates cross-ring fragmentation to obtain valuable monosaccharide linkage information. Our workflow has been adapted from standard proteomics-based workflows and, therefore, provides opportunities for laboratories with expertise in proteomics to acquire glycomic data with minimal deviation from existing buffer systems, chromatography media, and instrument configurations. Furthermore, our workflow does not require a mass spectrometer with high-resolution/accurate mass capabilities. The rapidly evolving appreciation of the biological significance of glycans for human health and disease requires the implementation of high-throughput methods to identify and quantify glycans harvested from sample sets of sufficient size to achieve appropriately powered statistical significance. The LC-MSn approach we report generates glycan isomeric separations and robust structural characterization and is amenable to autosampling with associated throughput enhancements.

## Highlights

• LC separation of N- and O-linked glycans as their permethylated derivatives.
• Glycan isomers resolved on reversed phase columns commonly used for peptides.
• Analytes identified by exact mass and retention time relative to glycan standards.
• Structural topologies validated by on-the-fly MS^n^ using accessible instrumentation.

## Graphical Abstract

**Figure:**
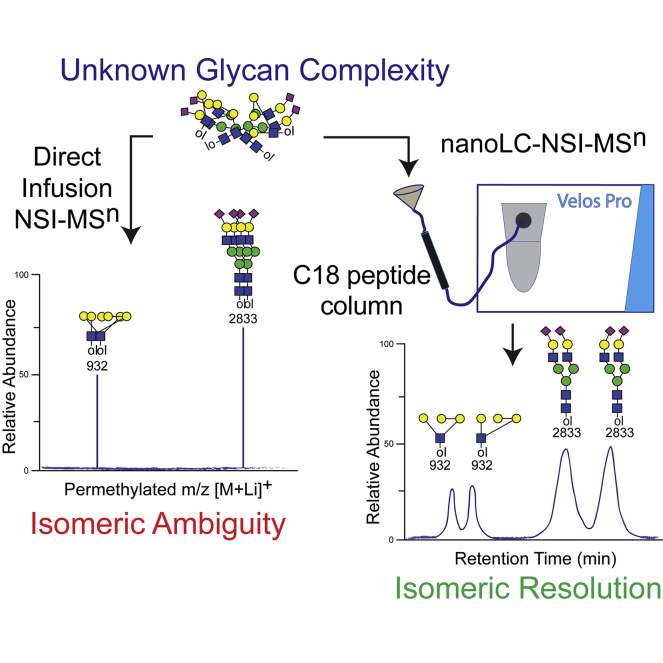

## In Brief

Structural analysis of glycoprotein N-linked and O-linked glycans is greatly enhanced by permethylation following release from the protein backbone. HPLC of permethylated glycans resolves isomeric glycans that are difficult to discern by direct infusion methodologies. An approach is described that utilizes standard peptide separation columns and accessible instrumentation. The methodology is easily adoptable by laboratories already familiar with LC-based peptide separations to achieve isomeric resolution of glycans released from biological materials.

Every living cell is covered in a dense and complex array of glycoconjugates that modulate various biological processes such as molecular recognition, cell–cell, and cell–matrix interactions. The structural diversity of glycans arises from differences in monosaccharide compositions, anomeric confirmation, glycosidic linkages, branching, and their attachment to protein or lipid (1). A hallmark of protein glycosylation is the phenomenon of microheterogeneity or the property that any single glycosylation site may be unmodified or modified by more than one glycan structure when comparing molecules of the same protein, even if produced in the same cell type. This diversity presents unique challenges for characterizing glycoprotein glycosylation and creates vital needs for technologies that robustly capture glycan structural details (2). The biochemical and analytical tools currently deployed for glycomic analysis are almost as diverse as the biological roles of glycans and are highly influenced by the research purpose.

Given the complexity of glycan structures, with special reference to the broad range of isomeric possibilities, glycan separation by LC has emerged as a state-of-the-art approach in glycomics. Almost all previous LC work has been done on underivatized or fluorescently tagged glycans, allowing high sensitivity of detection and high resolution separations based on size, charge, and hydrophobicity (3). These approaches require previously characterized glycan standards as reference materials or sequential enzymatic (*e.g.*, exoglycosidase sequencing) and/or chemical treatments (*e.g.*, hydrofluoric acid) for complete glycan characterization. While offline or online MS-based methods can be combined with tagged-glycan separations, the sensitivity and information content of the resulting data is significantly influenced by the ionization characteristics and chemical nature of the tag. In that respect, permethylation has emerged as a routine derivatization procedure that improves sensitivity by enhancing MS ionization. Glycan permethylation converts the oxygen of all hydroxyl groups, including the sialic acid carboxylates, into an O-methyl groups (O-CH_3_). Because glycans generally have a large number of free hydroxyls, the O-methyl groups of permethylated glycans dominate the chemical behavior of the molecule, enhancing and equalizing ionization across diverse glycan structures (4). Permethylation thereby allows for the analysis and quantification of acidic and neutral structures in the mass spectrometer's positive ion detection mode.

In sequential fragmentation MS/MS and MS^n^ analysis, “scars” of a free hydroxyl group instead of O-methyl are detected at the original position of glycosidic linkages, thereby providing valuable linkage information through cross-ring cleavages. Permethylation and heavy metal cations facilitate these cross-ring fragments enabling in-depth structural characterization of glycans by direct infusion into the mass spectrometer without LC separation (5, 6, 7, 8). However, structural elucidation of isomeric permethylated glycans poses analytic challenges in direct infusion MS because they are detected at the same m/z. This complication can be conquered by combining LC separation of permethylated glycans with intelligent MS^n^ in realtime. Various LC-MS methods for analyzing permethylated glycans have been published recently (9, 10). Here, we report our solution, which is designed to be rapidly adaptable by laboratories familiar with standard proteomic workflows. Using an Ultimate 3000 RSLC HPLC system coupled to a Velos Pro Dual Pressure Linear Ion Trap, we describe parameters of sample preparation, mobile phase and gradient optimization, as well as postacquisition data analysis of permethylated chemically synthesized standards and glycans released from either standard proteins or biological sample mixtures. We have achieved baseline separation and MS^n^ of isomeric N- and O-glycan structures to aid in confident identification and quantification by pushing the ionization into a single adduct form, namely lithium. Our workflow simplifies postacquisition data analyses and enhances throughput of glycomic data collection without drastically changing chromatography media used for proteomics or requiring a mass spectrometer with high-resolution/accurate mass capabilities.

## Experimental Procedures

### Materials and Reagents

PNGase F (N-glycanase) was obtained from the Complex Carbohydrate Research Center, University of Georgia, (Dr Kelley Moremen). PNGase A was acquired from NEB. N-Glycan standards were obtained from the Consortium for Functional Glycomics (CFG) and from The Scripps Research Institute (Dr James Paulson). O-Glycan standards were obtained as part of an NIH Common Fund grant (R21AI123161) from the University of Georgia (Dr Christopher M. West). Sodium hydroxide (NaOH) (50%) was purchased from Fisher Scientific. Sep-Pak C18 disposable extraction columns were obtained from Waters Corporation. AG-50W-X8 cation exchange resin (H^+^ form) was purchased from Bio-Rad, and trifluoroacetic acid from Pierce. Malto-series oligosaccharides were obtained from Wako Chemicals. Trypsin, Chymotrypsin, bovine pancreatic ribonuclease B (RNaseB), Fetuin, Dextran (Leuconostoc spp., Cat# 31388), and all other chemical reagents were purchased from Sigma-Aldrich. Fly powder, a dried preparation of *Drosophila* embryo glycoproteins, was prepared as previously described and used as a source of pauci-mannose glycans (11). Mouse brain extracts were prepared as previously described for analysis of brain O-glycans (12). HIV gp120 was recombinantly expressed in glyco-engineered *Pichia pastoris* strains obtained from GlycoFi to generate proteins with restricted glycan diversity (13, 14).

### Preparation of protein-rich powder and subsequent O-glycan release

Primary mouse tracheal epithelial cells (mTECs), mouse brain (Pel-Freez Biologicals), or HEK293 cells (kind gift from Dr Henrik Clausen, University of Copenhagen, Denmark) were homogenized in ice-cold 50% (v/v) aqueous methanol and delipidated with chloroform/methanol/water (4:8:3, v/v/v) as described previously (11, 15). Insoluble proteins were precipitated by centrifugation, and protein pellets were washed twice with ice-cold acetone before drying under a gentle Nitrogen stream to produce fine protein powder. Two to 3 mg of protein powder was then subjected to reductive β-elimination, and the released O-glycan alditols were purified as described previously (16). Briefly, protein powder was resuspended in 100 mM NaOH containing 1 M sodium borohydride and incubated for 18 h at 45 °C in a glass tube sealed with a Teflon-lined screw top. After incubation, the reaction mixture was neutralized with 10% acetic acid (AcOH) on ice and desalted using a AG-50W-X8 (H^+^ form) column (1 ml bed volume) before borate removal and Sep-pack C18 cartridge clean-up.

### Release and reduction of N-glycans

Preparation of glycopeptides and release of N-glycans was performed as described previously (11). Briefly, 20 to 75 μg of glycoproteins (RNAse B, Fetuin, gp120) were dried and resuspended in trypsin buffer (0.1 M Tris-HCl, pH 8.2, containing 1 mM CaCl_2_) by sonication and boiled for 5 min before addition of trypsin and chymotrypsin solutions. After incubation for 18 h at 37 °C, the (glyco)peptide mixture was boiled for 5 min and adjusted to 5% AcOH before a Sep-Pak C18 cartridge column clean up. Glycopeptides were eluted stepwise in 20% isopropyl alcohol in 5% AcOH and 40% isopropyl alcohol in 5% AcOH. Both, the 20 and 40% isopropyl alcohol fractions were pooled and evaporated to dryness. Dried glycopeptides were resuspended in 25 mM sodium phosphate buffer, pH 7.5, for digestion with PNGaseF (fetuin, ribonuclease B) or in 50 mM ammonium acetate buffer, pH 4.5, for digestion with PNGaseA (fly powder) before incubation for 18 h at 37 °C. PNGase-released oligosaccharides were separated from residual (glyco)peptides by another round of Sep-Pak C18 cartridge clean-up.

For the reduction of PNGase-released N-glycans and standards, dried glycans were resuspended in 100 μl of 50 mM NaOH, vortexed, and ultrasonic water bath sonicated before the addition of 100 μl 2% (w/v) sodium borohydride in 50 mM NaOH. After incubation for at least 4 h at RT, 200 μl of 10% (v/v) AcOH were added to neutralize the reaction. Samples were dried down under a gentle nitrogen stream before borate removal by adding 400 μl of 10% (v/v) AcOH in methanol and repeated evaporation (up to three times).

### Permethylation

All released N- and O-linked glycans as well as standards were permethylated before MS analysis according to the method by Anumula and Taylor (17). Dextran, malto-series oligosaccharide standards and CFG N-glycan standards were permethylated with ^13^C methyliodide (^13^C-MeI).

### nanoLC-MS/MS of permethylated glycans

Dried permethylated glycans and standards were dissolved in 100% methanol, and an aliquot (usually 5–10% of the total sample) was combined with an internal standard mix (^13^C-permethylated degree of polymerization [DP]4) and mobile phase A. For each nanoLC-MS/MS analysis, 3 to 6 μl of the prepared sample mix was injected for LC separation at 60 °C and a constant flow rate of 300 nl/min using an Ultimate 3000 RSLC (Thermo Fisher Scientific/Dionex) equipped with a PepMap Acclaim analytical C18 column (75 μm × 15 cm, 2 μm pore size) coupled to a Thermo Fisher Scientific Velos Pro Dual-Pressure Linear Ion Trap mass spectrometer (ITMS), ending with sample ionization *via* a stainless steel emitter. The three solvent systems used were either 0.1% formic acid in water for mobile phase A and 0.1% formic acid in 80% acetonitrile (ACN) for mobile phase B (“Proteomics buffers”); 0.1% AcOH containing 0.1 mM lithium acetate (LiOAc) for mobile phase A and 0.1% AcOH in 80% ACN containing 0.1 mM LiOAc for mobile phase B (“Low lithium buffers”); or 0.02% AcOH containing 1 mM LiOAc for mobile phase A and 0.02% AcOH in 80% ACN containing 1 mM LiOAc for mobile phase B (“High lithium buffers”).

For O-glycans, after equilibrating the column in 99% mobile phase A for 5 min, separation was achieved using a linear gradient from 30% to 70% mobile phase B over 150 min. For N-glycans, after equilibrating the column in 99% mobile phase A for 5 min, separation was achieved using a linear gradient from 45% to 70% mobile phase B over 150 min. The analytical column was regenerated after each run by ramping to 99% mobile phase B for 10 min and then returning to 99% mobile phase A to re-equilibrate.

The Velos Pro Dual Pressure Linear ITMS was operated in positive ion mode with a spray voltage of 1.8 to 2.2 kV and capillary temperature set at 210 °C. The MS method consisted of first collecting a Full ITMS (MS1) survey scan in profile mode and m/z mass range from 500 to 2000 with automatic gain control Target set to 30,000.00, followed by data-dependent MS2 fragmentation of the top 3 to 5 most intense peaks using collision-induced dissociation at 40 to 42% collision energy and an isolation window of 2 m/z. Dynamic exclusion parameters were set to exclude ions for fragmentation for 15 s if they were detected and fragmented five times in 15 s. For targeted MS^n^ approaches, intelligent fragmentation methods were generated using neutral loss function and product dependent MS^3-5^ acquisition to determine sialic acid linkage of terminally sialylated N-glycan structures. To normalize elution times on the RP-column in terms of g.u., ^13^C-permethylated dextran (4–10 g.u.) was analyzed before and after a sequence of samples. After the completion of a sample set, in preparation for limited HPLC use for an extended period of time, the postcolumn-fused silica lines were manually flushed with 50% methanol in water to avoid clogging of the stainless steel emitter with lithium salts. All data were processed manually using the Xcalibur software package 2.0. GRITS Toolbox; a freely available tool for semiautomated annotation of glycomic MS data was also employed to generate candidates for structural assignment (18). The GRITS Toolbox parameters used for annotations were set as follows: all glycosidic and cross-ring cleavage types enabled (B, Y, C, Z, A, X); maximum number of cleavages and cross-ring cleavages, 2; mass type, average; mass accuracy for full MS, 1 Da; mass accuracy for MS^n^, 250 ppm; precursor intensity cut-off, 5%; database, “N-glycans (topology)” with prefilter human monosaccharides enabled; reducing end, reduced; PerDeriv type, perMe; derivative mass and adduct were set according to experimental conditions, isotopically heavy methyl (^13^C-MeI) or Na/Li as appropriate. Raw mass spectrometry data files have been deposited at GlycoPOST (https://glycopost.glycosmos.org/) under the Accession ID number GPST000123.

## Results

### Sample preparation

**Fig. 1:**
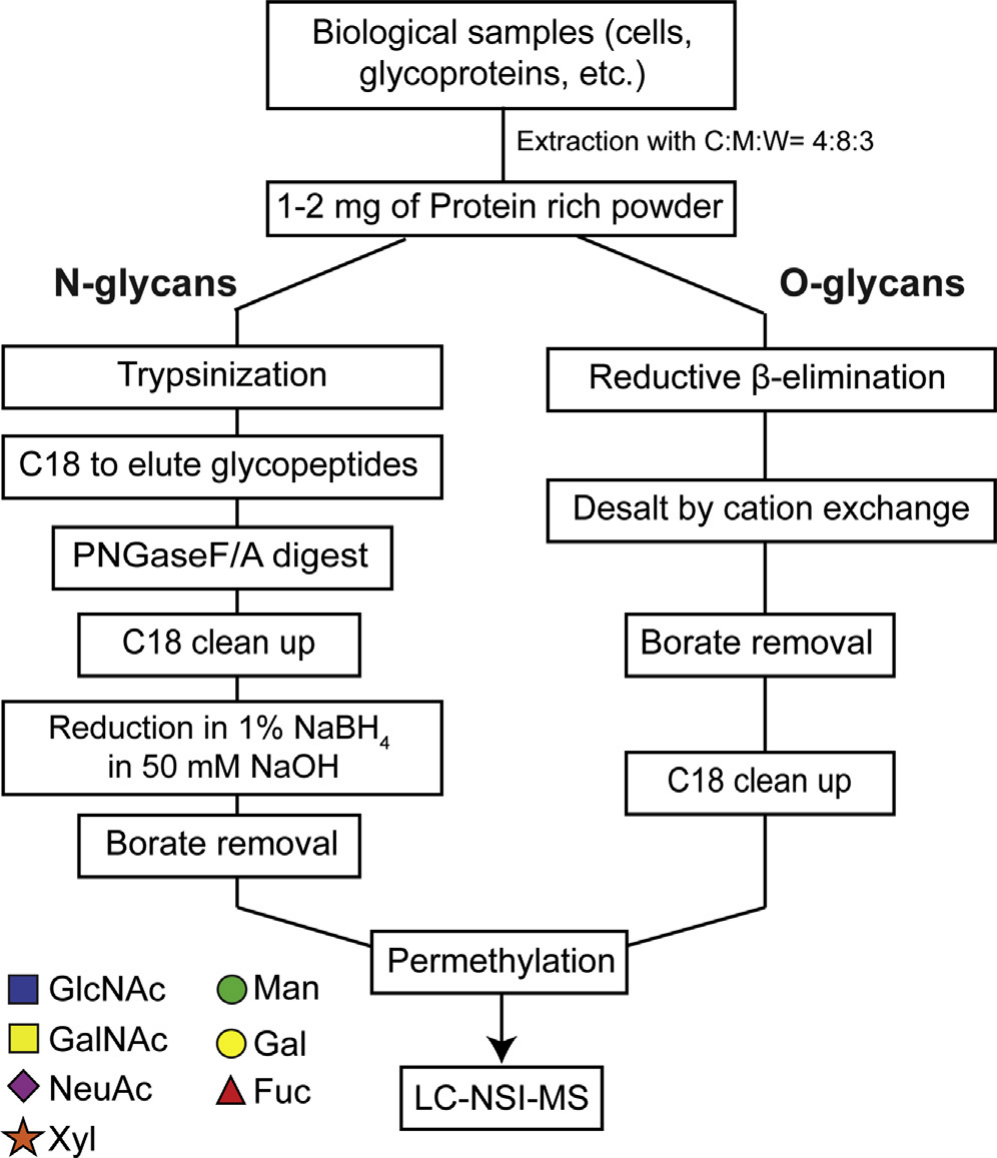
**General workflow for preparation of N- and O-glycans.** Using virtually any biological source as starting material, protein-rich powder can be generated *via* organic delipidation as described previously. N-linked glycans can be released enzymatically (PNGase F or PNGase A) followed by mild reduction of the free reducing terminus. O-linked glycans can be released chemically *via* reductive β-elimination. Released glycans of either class are subjected to permethylation followed by direct analysis by LC-NSI-MS. Symbol and Text nomenclature for representation of glycan structures is displayed according to the Symbol Nomenclature for Glycans (SNFG) (39). LC-NSI-MS, liquid chromatography-nanospray ionization-mass spectrometry.

Our workflow can be applied to any biological starting material. In the first preparation steps, glycosphingolipids are extracted from the biological sample before acetone precipitation to obtain protein rich powder (Fig. 1). Starting with 1 to 2 mg of protein powder each, we can prepare N- and O-glycans. For N-glycans, the glycoproteins are proteolytically cleaved into glycopeptides (usually by trypsin and/or chymotrypsin) and purified using a C18 solid-phase extraction column. N-glycans are then enzymatically released with PNGase F. For liquid chromatography-nanospray ionization-mass spectrometry (LC-NSI-MS) analysis, released N-glycans can also be reduced in mild sodium borate conditions before borate removal and permethylation for improved chromatographic separation (see supplemental Fig. S1). O-glycans are chemically released by reductive β-elimination, followed by desalting, borate removal, and C18 clean up. Following permethylation and phase partitioning, we subjected the organic phase, which contains neutral and nonsulfated glycans, to analysis by LC-NSI-MS using various buffer and gradient conditions.

### O-glycan separation and mobile phase optimization

**Fig. 2:**
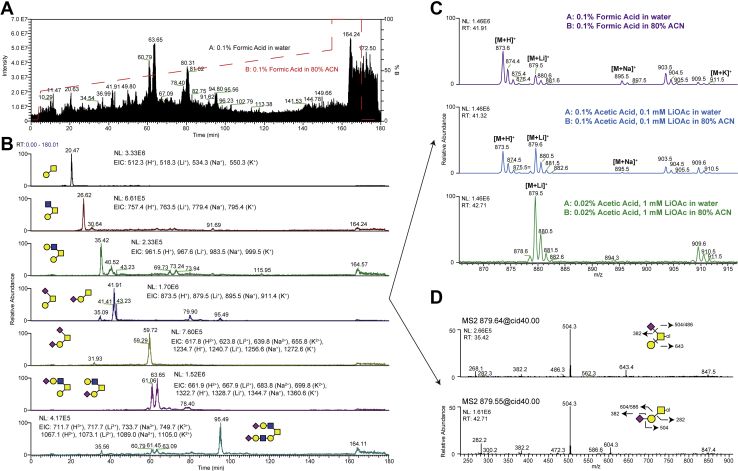
**Addition of lithium acetate to LC-mobile phases enhances ionization.** Reduced O-glycans from mouse tracheal epithelial cells (mTECs) were separated on a PepMap Acclaim C18 column (75 μm × 15 cm, 2 μm pore size) using three different mobile phase pairs (Buffers A and B) on a Dionex UltiMate 3000 coupled to Thermo Velos Pro ITMS. *A*, the total current chromatogram and extracted ion chromatograms (*B*) demonstrate baseline separation of permethylated O-glycans using common proteomics buffers (Formic acid/Acetonitrile). *C*, observed alkali metal adduct ions for the three mobile phase conditions tested demonstrated optimal ionization and adduct formation in 0.02% acetic acid containing 1 mM lithium acetate. *D*, MS/MS fragmentation of two glycan isoforms are shown.

To test the separation of permethylated O-glycans using our platform, initial RP C18 nanoLC-separations were performed on O-glycans released by reductive beta-elimination from primary mTEC. These initial separations used conventional acidic proteomic mobile phases at an elevated column oven temperature (60 °C) to facilitate baseline separation (Fig. 2*A*). As has been previously described and also in our analysis, lower column oven temperatures (40 °C and 50 °C) resulted in significant peak broadening (10). By generating extracted ion chromatograms (EICs) of expected glycan species, the major mTEC O-glycans were resolved, and multiple EIC peaks were identified for some, revealing the possibility of isomeric structures (Fig. 2*B*).

One disadvantage of using acidic mobile phases that are standard for proteomic analyses is the formation of multiple alkali metal glycan adducts which makes data analysis and, importantly, quantification more difficult. As demonstrated for a simple sialyl Tn O-glycan structure, multiple ions including protonated [M + H]^+^, lithiated [M+Li]^+^, sodiated [M+Na]^+^, and potassiated [M + K]^+^ adducts were detected in a Full MS scan using conventional proteomics mobile phases (Fig. 2*C*). To promote the ionization into only one molecular ion species and to enhance cross-ring cleavages, LiOAc was supplemented in the mobile phases at 0.1 mM resulting in an improvement but not complete shift toward lithiated ion species. By increasing the concentration to 1 mM LiOAc and reducing acid concentration, adduct ions were shifted to essentially lithiated-only forms (Fig. 2*C*). Combining EICs with the data-dependent MS/MS data acquisition in real-time, the structures of two isomeric sialyl Tn O-glycans were resolved with the minor branched structure eluting earlier (35 min) than the more abundant linear structure (42 min) (Fig. 2*D*).

### N-glycan separation and gradient optimization

To assess the feasibility of separating permethylated N-glycans, we used PNGaseF-released N-glycans from RNAseB as our glycan source. Initial peak-splitting during the LC-separation was observed, presumably because of mutarotation of the anomeric configuration at the free reducing end before permethylation (α- *versus* β-anomer; supplemental Fig. S1*A*). To ameliorate this complication, reduction of PNGase-released N-glycans before permethylation is strongly recommended for simplifying glycans to elute as one peak rather than two α- and β-anomer peaks (supplemental Fig. 1*B*). Peak splitting was not observed in our mTEC O-glycan analysis because the reducing termini of the released O-glycans are reduced during the release by reductive β-elimination as part of our standard workflow (Fig. 1).

**Fig. 3:**
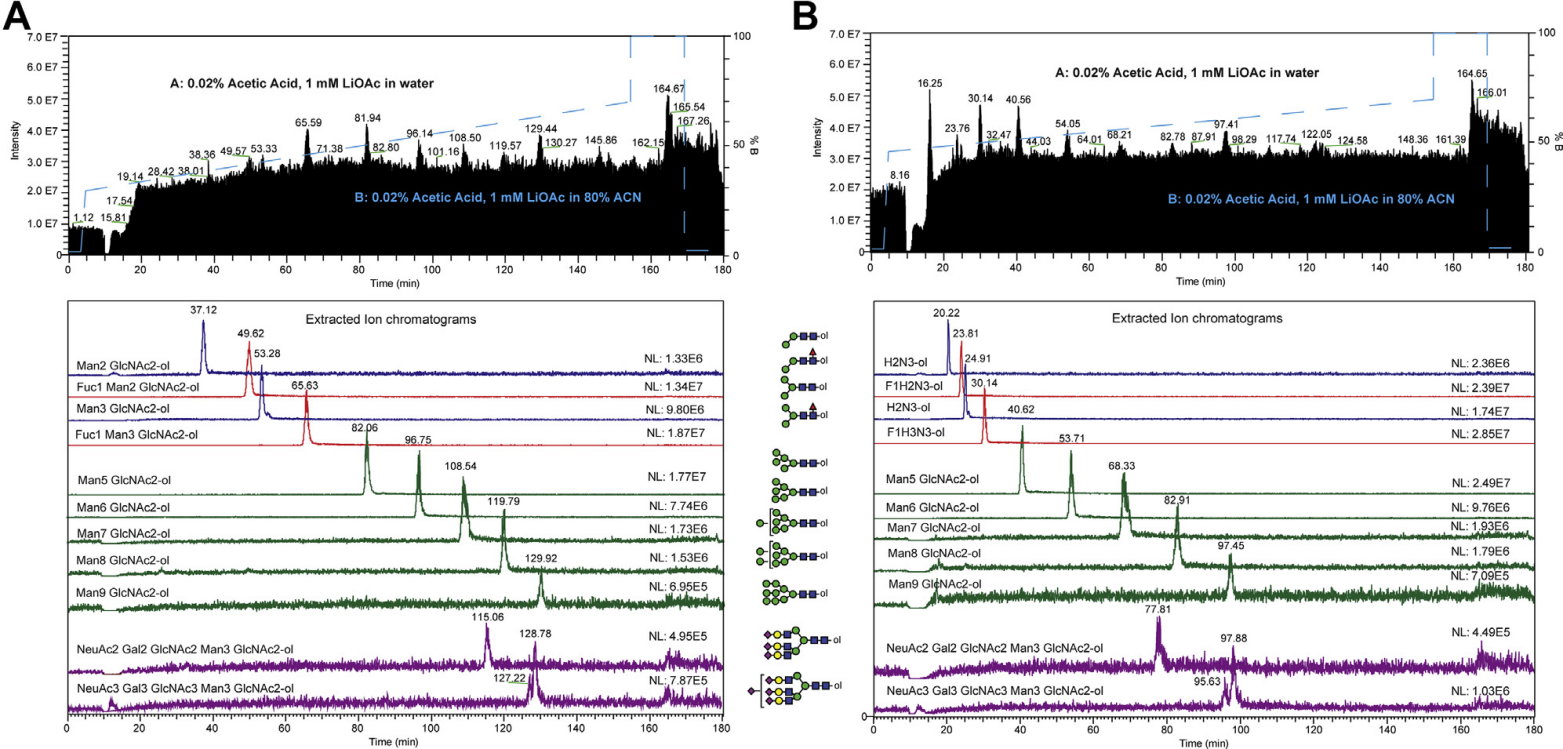
**LC-gradient optimization for reduced N-glycans.***A*, initial separation of paucimannose, high-mannose, and complex-type N-glycans was achieved with a gradient from 30 to 70% B, but separation of larger N-glycans (*e.g.*, tetra-antennary) might be insufficient. *B*, Rapid increase in the gradient to 45% B in 5 min followed by a shallow gradient from 45 to 70% B seems to be more suitable for larger N-glycans (*e.g.*, tri/tetra-antennary). Attempts to shorten the gradient program to less than 180 min using steeper gradients resulted in suboptimal separation of glycans and co-eluting species.

Using an initial separation gradient of 30% to 70% mobile phase B over 150 min, baseline separation was achieved for reduced and permethylated paucimannosidic, high-mannose, and complex type N-glycan structures released from fly protein powder, RNAseB, and Fetuin, respectively (Fig. 3*A*). As this gradient might be insufficient for separation of larger N-glycans such as tetra-antennary structures, a modified gradient with a rapid increase in the first 5 min to 45% B followed by a shallow gradient of 45% to 70%B over 150 min was applied and was observed to not change the overall elution pattern of the tested N-glycans (Fig. 3*B*). Using our platform and elution conditions, attempts to shorten the gradient to less than 180 min as described by others resulted in suboptimal separation of glycans and increased numbers of co-eluting species (3, 9, 10).

### Chromatographic retention times can be converted to glucose units

**Fig. 4:**
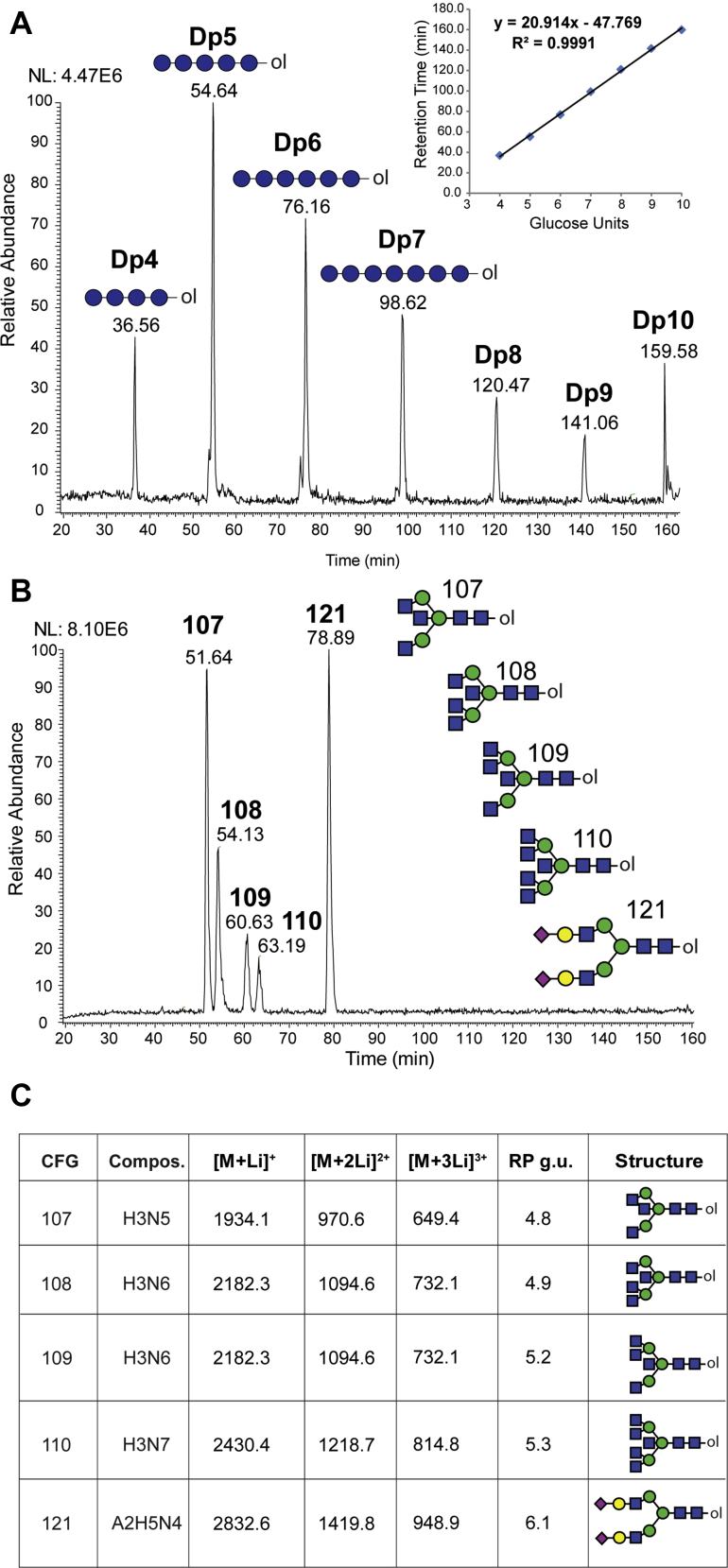
**Elution of permethylated glycans expressed in terms of glucose units**. *A*, reduced and ^13^C-permethylated Dextrans (isomaltooligosaccharide series, DP4 to 10) were separated using a PepMap Acclaim C18 column in 1 mM lithium acetate-containing mobile phases (45–70% B over 150 min). Extracted ion chromatogram for each predicted mass is shown (within 10 ppm). Retention times of DP standards, run both before and after sample acquisition queue, were averaged and plotted using a linear regression fitting. Error bars represent standard deviation (See *inset*). *B*, reduced and ^13^C-permethylated CFG N-Glycan standards (107–110, 121) were analyzed as described in (*A*). *C*, summary table of analyzed CFG standards with retention times converted to reversed phase glucose units (RP g.u.) using linear regression fitting shown in *A*, *inset*. CFG, Consortium for Functional Glycomics; DP, degree of polymeriation.

In many instances, the combination of LC retention time and accurate mass of a detected analyte can lead to structural assignment by reference to well-characterized standards. However, assignment/annotation solely based on retention time and mass is frequently impractical because of technical challenges associated with variability in column performance, clogged tubing, clogged emitters, etc. Therefore, a more robust relationship between glycan structure and retention time can be achieved by referencing glycan elution positions to a hexose polymer ladder, yielding retention times in glucose units or g.u (19, 20). Dextran polymers ranging in length (DP) from 4 to 10 monosaccharide units where reduced and permethylated with ^13^C-MeI to produce a dextran reference ladder of g.u. standards (DP4-10). Aliquots of the reference ladder were injected before and after sample acquisition queues, and the resulting retention times were averaged from EICs for each predicted mass within 10 ppm (Fig. 4*A*).

To test the robustness of our method for characterizing the elution positions of glycans with greater complexity than the dextran ladder, we analyzed four CFG standards (107, 108, 109 and 110) which represent bisected, complex branched N-glycans and one CFG standard (121), a sialylated biantennary N-glycan. Separation of all five reduced and ^13^C-permethylated CFG standards was achieved resulting in standard 107 (bisected, biantennary N-glycan) eluting before standard 108 (bisected, triantennary N-glycan with two GlcNAc residues attached to the core α1,3 mannose). Interestingly, a gap of 6 min in retention time was detected between the elution of the isomeric structure of standard 108, which is standard 109 (bisected, triantennary N-glycan with two GlcNAc residues attached to the core α1,6 mannose) demonstrating the power of our modified gradient to achieve separations based on size and hydrophobicity (Fig. 4*B*). CFG standard 110 (bisected, tetra-antennary N-glycan) eluted approximately 2.6 min after standard 109 and before the final sialylated, biantennary N-glycan standard 121. Using the linear regression fitting from the dextran ladder runs before and after the sample queue, the observed retention times were converted to reverse phase glucose units (RP g.u.) to express the elution positions of the analyzed CFG standards (Fig. 4*C*). Dextran ladder was analyzed before a large sample acquisition queue and subsequently after 25 days of continuous operation (supplemental Fig. S2). Retention times of isomaltose polymers after nearly 1 month of continuous use were consistent within 2 to 3%. Furthermore, retention times were consistent regardless of amount injected, in a dynamic range of 15.6 to 1000 fmol of injected standard (supplemental Fig. S3).

**Fig. 5:**
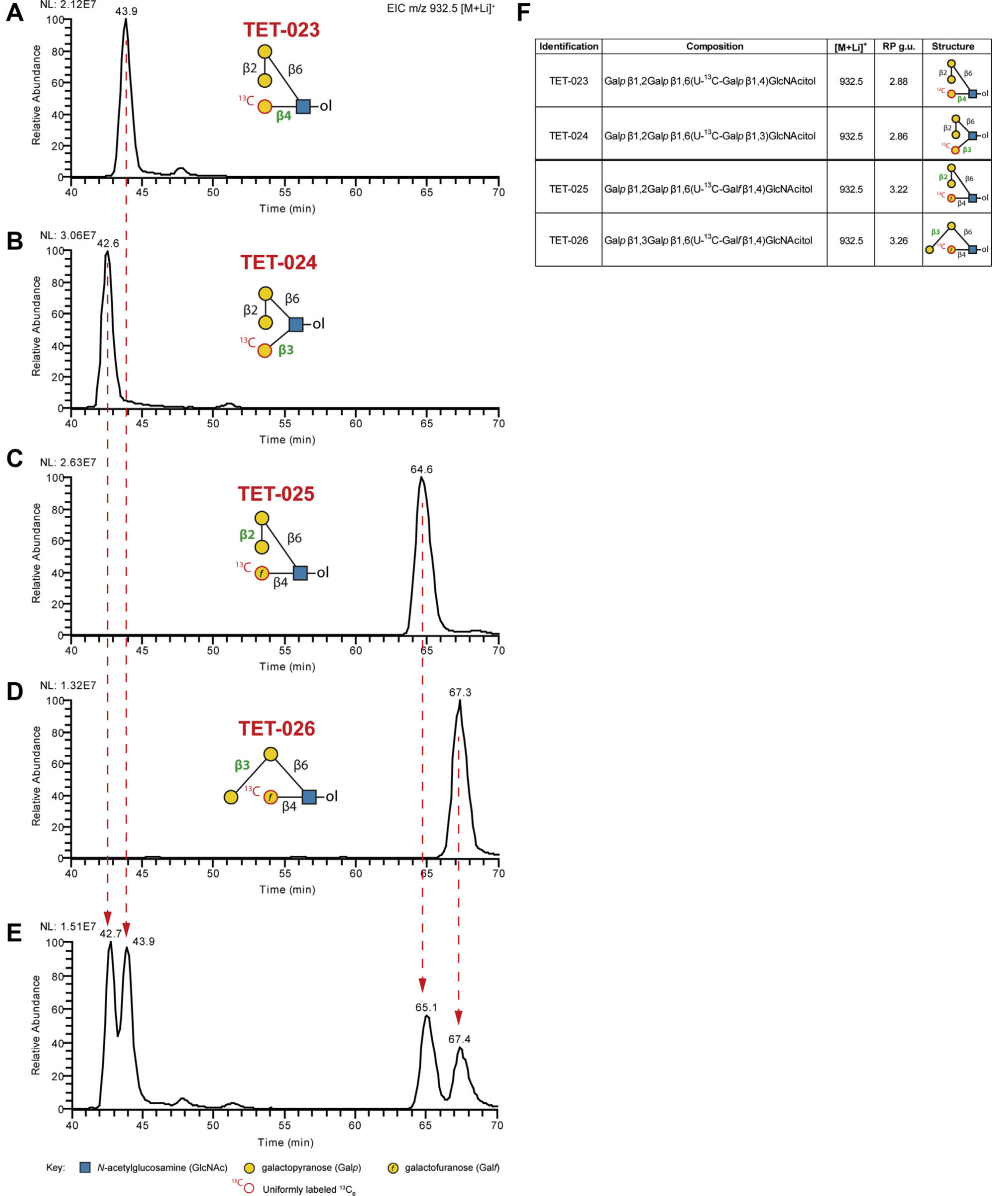
**LC-NSI-MS of Permethylated Isomers.***A*, extracted ion chromatograms showing individual (*A–D*) or a mixture (*E*) of four O-glycan tetrasaccharide isomers that only differ by the cyclic form of a single sugar [pyranose (p) or furanose (f)] or a single linkage (differences indicated in *green*). Separation was performed using a PepMap Acclaim C18 column in 1 mM lithium acetate-containing mobile phases with a modified gradient (35–40% B over 170 min) and detected using a Thermo Velos Pro ITMS. *F*, summary table of isomers used with retention times converted to reversed phase glucose units (RP g.u.; referenced to ^13^C-permethylated isomaltooligosaccharide standards separated using the same gradient). LC-NSI-MS, liquid chromatography-nanospray ionization-mass spectrometry.

Similar to the N-glycan standards, we tested the ability of our method to separate chemically synthesized, reduced, and ^13^C-permethylated O-glycan isomeric standards derived from the human pathogen *Trypanosoma cruzi*, the causative agent for Chagas disease in South America (21, 22). The four tetrasaccharide O-glycan isomers analyzed only differ by either the cyclic form of a single monosaccharide (pyranose or furanose) or a single linkage. Owing to the very small differences in isomeric confirmation between these four monosaccharides, a modified gradient was applied (35–40% B over 170 min) (Fig. 5). The four standards were analyzed separately (Fig. 5, *A*–*D*) or as a mixture (Fig. 5*E*) and demonstrated nearly baseline separation using the modified gradient program. Of the four isomeric O-glycan structures, the pyranose structures eluted over 20 min earlier than furanose-containing isoforms. As described previously, the observed retention times were converted to RP g.u. using the linear regression fitting from dextran ladder runs before and after the sample queue using the same gradient program (Fig. 5*F*).

### Characterization of N-glycans released from recombinant HIV gp120 expressed in glyco-engineered P. pastoris

**Fig. 6:**
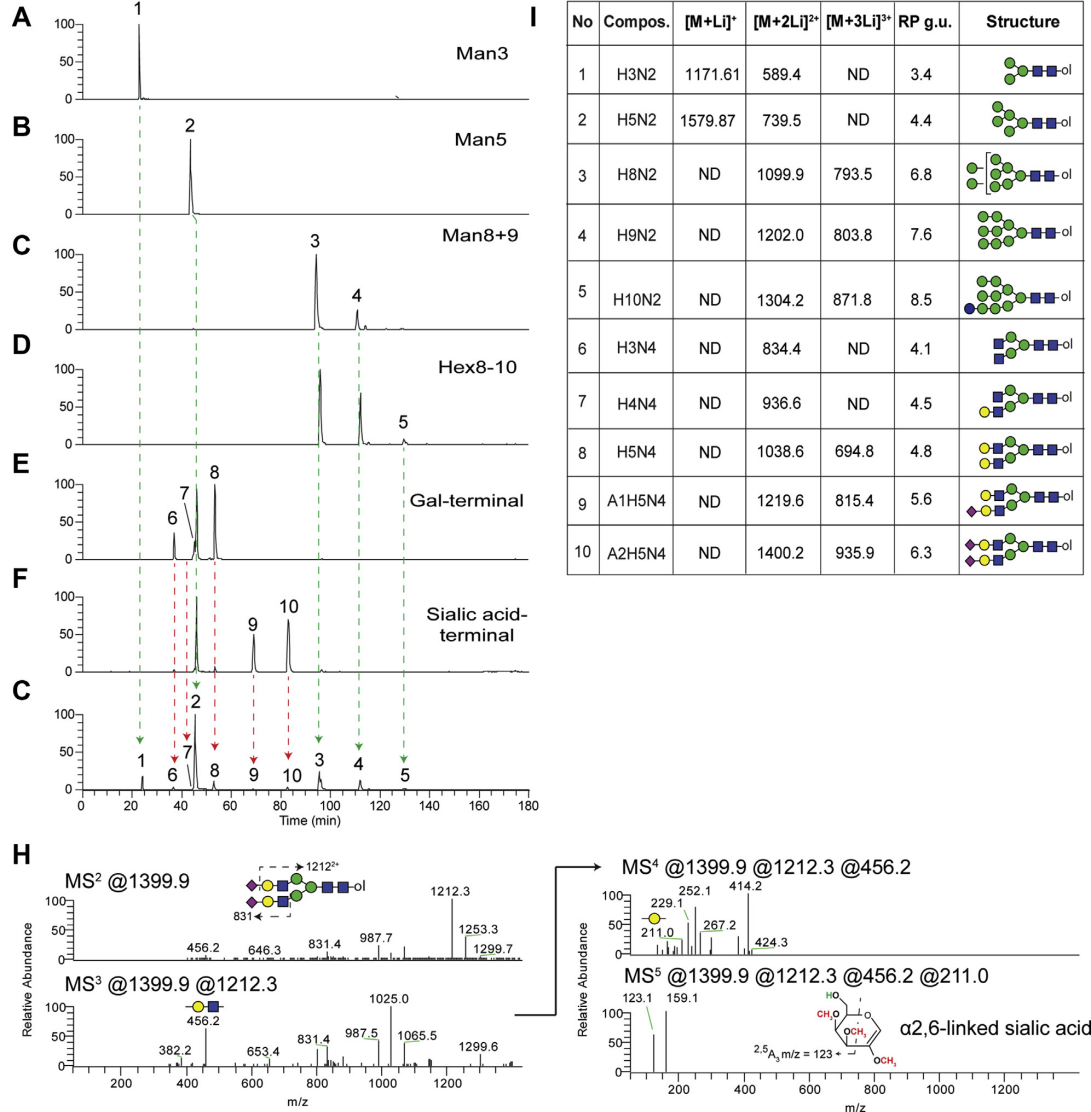
**LC-NSI-MS**^**n**^**analysis of N-glycans released from recombinant gp120 expressed in *Pichia pastoris* strains defective in N-glycosylation machinery.***A*, extracted ion chromatograms of all predicted PNGaseF-released, reduced, and permethylated N-glycans from various gp120 expressing cell lines, either individually (*A–F*) or as a mixture (*G*). *H*, automated, intelligent MS^n^ defines sialic acid linkage position in realtime from Structure #10. *I*, summary table of gp120 N-glycans with retention times converted to reversed phase glucose units (RP g.u.; referenced to ^13^C-permethylated isomaltooligosaccharide standards separated using the same gradient). LC-NSI, liquid chromatography-nanospray ionization; MSn, multidimensional ion fragmentation.

The data described above demonstrates the ability of our method to resolve N- and O-glycan structures derived from abundant glycoproteins or prepared as synthetic standards. To assess the applicability of our method for detecting biologically relevant glycosylation patterns, we analyzed N-glycans released from recombinantly expressed HIV gp120 envelope glycoproteins harvested from six different glyco-engineered strains of *P. pastoris* (13, 14). Each of these strains lack glycosyltransferase activities that result in the generation of restricted cellular N-glycan profiles predicted to be to predominated by specific high-mannose or complex type glycans. After release, reduction, and permethylation, N-glycans from each gp120 preparation were first separated and analyzed individually, demonstrating that the detected N-glycans are consistent with the targeted glycosyltransferases in each glyco-engineered strain (Fig. 6, *A*–*F*). For example, N-glycans of the composition Hex_3_HexNAc_2_-ol were only detected in the strain engineered to generate Man_3_GlcNAc_2_ structures on gp120 glycoprotein (Fig. 6*A*) and Hex_5_HexNAc_2_-ol in the Man_5_GlcNAc_2_ engineered strain (Fig. 6*B*). A similar pattern was observed in engineered strains producing only Man_8_GlcNAc_2_ and Man_9_GlcNAc_2_ as well as Hex_8_HexNAc_2_-Hex_10_HexNAc_2_ structures (Fig. 6, *C* and *D*). *Pichia* strains engineered to synthesize N-glycan structures with terminal galactose residues or terminal sialic acid modifications also exhibited Man_5_GlcNAc_2_ structures in addition to the expected complex type structures (Fig. 6, *E* and *F*). To assess the capacity of our LC-MS system to resolve the range of glycans released from these expression platforms, we mixed equal amounts of each sample and analyzed as described above. We were able to accurately detect and resolve all of the various N-glycan structures derived from the recombinant gp120 glycoforms that were detected when each were analyzed individually (Fig. 6*G*). Furthermore, we applied an automated MS^n^ workflow which targeted the neutral loss of permethylated sialic acid (Δm/z = 375) to trigger product dependent MS^3-5^ acquisitions. Because lithium was included in the LC separation mobile phases, this workflow revealed that the terminal sialic acids of the N-glycan structures released from these gp120 preparations are α2,6-linked (Fig. 6, *H* and *I*).

### Analysis of O-glycan isomers from biological samples

**Fig. 7:**
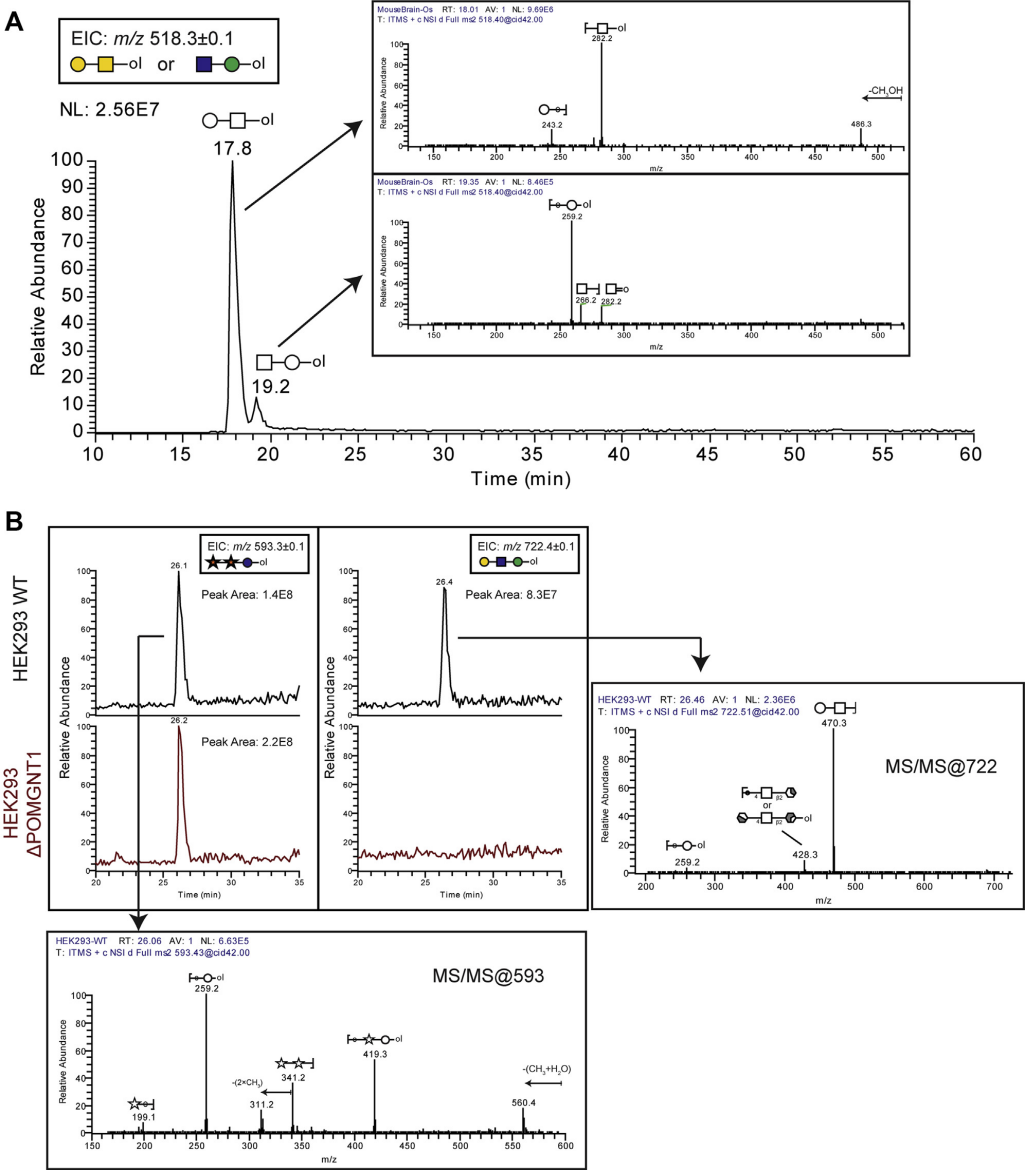
**Analysis of reduced and permethylated O-glycans released from mouse brain proteins and human cell lines defective in the O-mannosylation pathway.***A*, extracted ion chromatogram of Hex_1_HexNAc_1_ structures released from mouse brain proteins indicates two structural isomers. MS2 fragmentation of m/z 518.3 at the indicated retention times are shown as insets. *B*, LC-MS analysis of O-glycans released from HEK293 WT and ΔPOMGNT1 mutant cell lines. Respective EICs and annotated MS2 spectra are shown. EICs, extracted ion chromatograms.

O-linked glycans linked to Ser/Thr residues can be divided into various subtypes depending on the initiating monosaccharide linked directly to the polypeptide backbone. The most abundant class is the mucin-type O-GalNAc-initiated structures. Other types of O-glycans include O-Mannose, O-Fucose, O-Glucose, O-GlcNAc, O-Xylose, all of which have diverse biological functions (23, 24). Previous studies have documented the detection of both O-GalNAc and O-Mannose-initiated disaccharides and Galβ3GalNAc and GlcNAcβ2Man, respectively, in mouse brain (12, 25). In direct infusion MS, these glycans are detected at the same m/z because they have the same Hex_1_HexNAc_1_ composition. While MS/MS fragmentation of the permethylated disaccharides can detect the presence of these two glycans, the fragmentation patterns of the isolated ions can be complicated as they are a mix of the two species and quantification of each individual disaccharide is problematic. To identify the presence or absence of Hex_1_HexNAc_1_ isomers in mouse brain, we analyzed the released and permethylated glycans by our LC-MS workflow (Fig. 7*A*). An EIC of the expected singly charged, lithiated structures (*m/z* 518.3) revealed two peaks of differing relative abundance at 17.8 and 19.2 min. MS2 fragmentation of m/z 518 at the respective times is consistent with the HexNAc-initiated disaccharide (O-GalNAc) eluting earlier than the Hexose-initiated structure (O-mannose), as determined by unique fragments at the reducing terminus (Fig. 7*A*, insets).

We also analyzed O-glycans harvested from cells that were glyco-engineered to be deficient in the O-Mannosylation pathway implicated in congenital muscular dystrophies (26, 27, 28). The glycosyltransferase POMGNT1 (Protein O-Linked Mannose N-Acetyl-glucosaminyltransferase 1) transfers β2-linked GlcNAc to an underlying α-mannose linked to Ser or Thr to generate Core M1-type glycans which can be further extended by B4GALT1 with β4-linked galactose (29). O-glycans were released from glycoproteins harvested from HEK293 wildtype cells or HEK293 cells harboring a Crispr-Cas9-mediated global disruption of the POMGNT1 locus (30). As a control, a non-O-Mannose structure (Xylα3Xylα3Glc-ol), which modifies a consensus found in the EGF repeats of a relatively small number of proteins (31, 32, 33), was detected (m/z 593.3 [M+Li]^+^) in both samples (Fig. 7*B*). An EIC of m/z 722.1 ([M+Li]^+^) which corresponds to the composition Hex_2_HexNAc_1_ (O-mannose Core M1 structure, Galβ4GlcNAcβ2Man-ol) demonstrates the presence of the glycan in HEK293 WT but not in the POMGNT1-deficient cell line.

## Discussion

The field of glycomics has historically been eager to engage new analytic platforms based on their promise to deliver greater sensitivity and enhanced detection of structural features (34, 35, 36, 37). Despite tremendous advances in various analytic technologies, it remains true that no single technique, besides NMR, is capable of routinely and completely defining glycan structures. Thus, the field will continue to benefit from the development of an expanding array of analytic approaches. We have combined two powerful technologies, glycan permethylation and LC, in a manner that should be adoptable by analytic laboratories familiar with standard LC-MS/MS proteomic techniques. To facilitate adoption of our nanoLC-NSI-MS^n^ approach, we have optimized run conditions and buffer compositions for analysis of N-linked or O-linked glycans. Using these conditions, we achieved baseline separations of challenging isomeric glycans and also demonstrated the power of on-the-fly MS^n^ to distinguish structural features such as the linkage position of sialic acids.

Other investigators have previously described LC-based separations of glycans and, more particularly, of permethylated glycans (3, 9, 10). These approaches are all valuable contributions to the arsenal of glycomic technology. In many cases, these techniques make use of glycan standards to define the retention times of known structures, generating a database of RT values that, when combined with accurate mass detection, can provide confident identification of components in complex mixtures. Glycans with novel RT values can be at least partially characterized using these methods by applying off-line exoglycosidase digestions (sequencing) and re-analysis (37). Our approach is also amenable to off-line exoglycosidase digestion with re-analysis, takes advantage of RT and accurate mass, and will, over-time build a library of RP g.u. values to help define structural features of novel glycans. The resolving power of our method is most clearly demonstrated by its ability to generate near-baseline separation of four tetrasaccharides that share the same monosaccharide composition and topology but differ only in a single linkage position (see Fig. 5). To this resolving power, we add the capability of capturing MS^n^ data on-the-fly to help assign structures with novel RT values in a single analytic run (see Figs. 6 and 7). The inclusion of lithium in the mobile phase enhances this capability by facilitating the generation of cross-ring fragments indicative of specific structural features (5).

We optimized the lithium concentration in the mobile phase so that we detect glycans as a single adduct species. This simplification allows quantification to be done from peak areas detected in MS1 for a single adduct, enhancing sensitivity and resolution of glycan species across the chromatogram. As mentioned above, our method incorporates the inclusion of RT standards (DP4-10) which allows the calculation of relative retention times in Glc units (RP g.u.), facilitating cross-platform and run-to-run identification of glycans of interest. We have previously demonstrated that permethylated glycans yield nearly the same signal intensity regardless of their mass or complexity, unless modified with sulfate or phosphate (4). Thus, known amounts of DP standards can not only serve as RP g.u. references but may facilitate quantification of molar amounts of analyte. However, additional work is required to determine whether this approach can be validated as semiquantitative or can provide absolute quantitation across various classes of glycans. A current limitation of our approach is the need for well-defined glycan standards to fully characterize complex mixtures of unknown structures. Ideally, a standard would be available for every glycan in the analyte and, over time, extensive tables of RP g.u. values would be generated to facilitate rapid identification of components of interest. Confidence in the usefulness of RP g.u. tables populated by our approach will be heightened by the MS^n^ data that are generated to support structural features of standards or mixtures of standards.

Previously published glycomic studies from our laboratories and many others, more than can be reasonably cited, have relied heavily on direct infusion of permethylated glycans. These experiments, while valuable for in-depth structural analysis, require the presence of the operator for sample load, run, and subsequent wash before load of next sample. The nanoLC-NSI-MS^n^ method we describe here, like other LC-based methods, is amenable to autosampling, greatly increasing analytic throughput. Furthermore, data collection is almost entirely automated compared with more labor-intensive direct infusion experiments. This marked benefit also highlights a bottleneck in all glycomic analysis, but especially for LC-based analysis, namely, facile analysis of large data sets. Software platforms capable of handling and annotating large glycan data sets generated by nanoLC-NSI-MS^n^ approaches are only in their infancy (38). We made use of our in-house developed GRITS Toolbox to sieve through the data by providing structural candidates based on MS/MS data (18). However, neither this platform nor any other is currently capable of integrating MS^n^ data for permethylated glycans across an entire LC run. Furthermore, software packages developed for LC-based proteomic applications are not ideal for interpreting LC of permethylated glycans because the underlying assumptions regarding isotopic distributions of amino acids are not transferable to permethylated monosaccharides.

Fully automated annotation packages for handling permethylated glycan LC-separation data are a significant need for expanding the adoption of this powerful approach for glycomics. Here, we have provided a useful and robust separation platform that will facilitate the generation of data essential for developing and testing such tools. In the meantime, as these new software tools are developed and implemented, the expanded ability of our nanoLC-NSI-MS^n^ platform to resolve and quantify glycan isomers, combined with the capacity for high-throughput analysis, will provide new opportunities for investigating the diversity of glycosylation at scales capable of answering biologically important questions.

## Data availability

Raw mass spectrometry data files have been deposited at GlycoPOST (https://glycopost.glycosmos.org/) under the accession ID number GPST000123.

## Conflict of interest

The authors declare no competing interests.
