# Supplementary material for: Separation and Identification of Permethylated Glycan Isomers by Reversed Phase NanoLC-NSI-MSn
Source: Mol Cell Proteomics. 2021 Jan 26;20:100045. doi: 10.1074/mcp.RA120.002266 (PMC8724860; doi:10.1074/mcp.RA120.002266)
Supplement: Supplemental Figures S1 to S3 [file mmc1.pdf]

Supplemental Material

Separation and identification of permethylated glycan isomers by reversed phase nanoLC-NSI-MS^n

Simone Kurz[1,*], M. Osman Sheikh[1,*], Shan Lu[3], Lance Wells[1,2,†], and Michael Tiemeyer [1,2,†]

Supplement 1 - Reduction of N-glycans

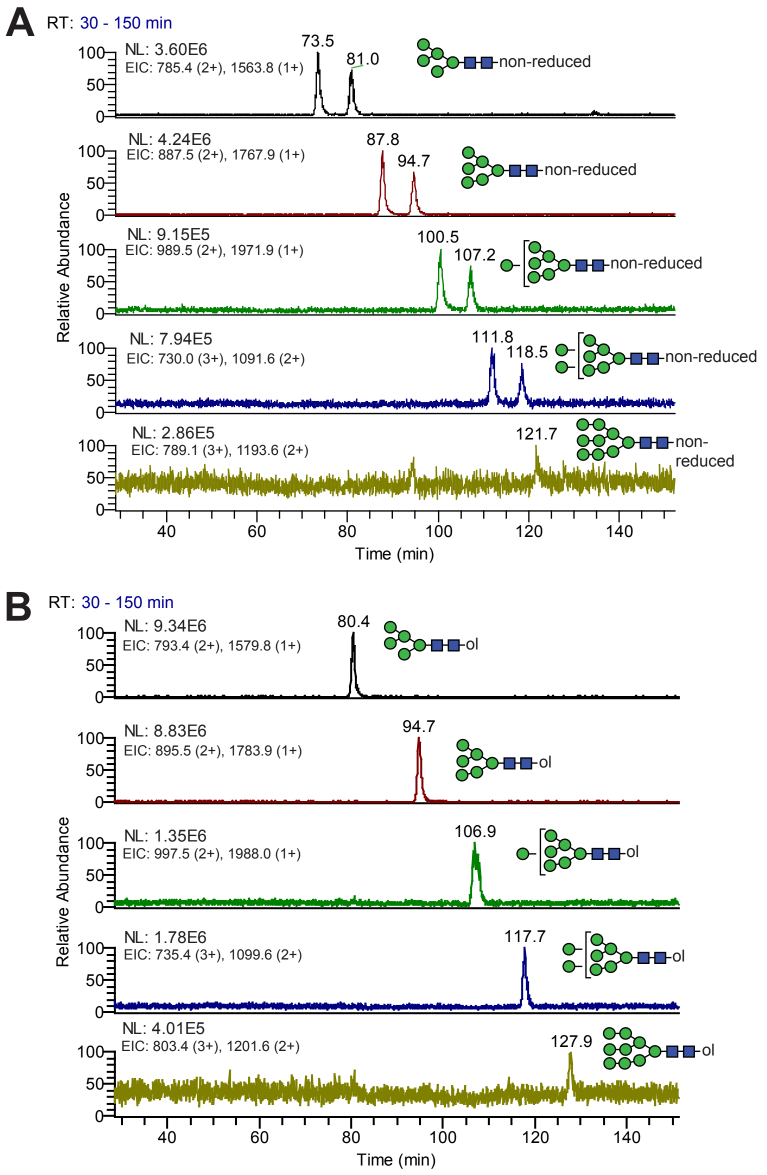

Supplement 1. Reduction of N-glycans prior to permethylation is recommended to avoid peak-splitting during LC-separation. PNGase F released N-glycans from RNAse B were subjected to PepMap Acclaim C18 LC separation in 1 mM lithium acetate-containing mobile phases and detected using Thermo Velos Pro MS. Extracted Ion chromatograms of predicted RNAse B permethylated Man5-Man9 structures demonstrate that non-reduced N-glycans tend to peak-split due to the anomeric configuration (**A**), while reduced glycans do not (**B**).

Supplement 2 - Reproducibility of Standard Performance

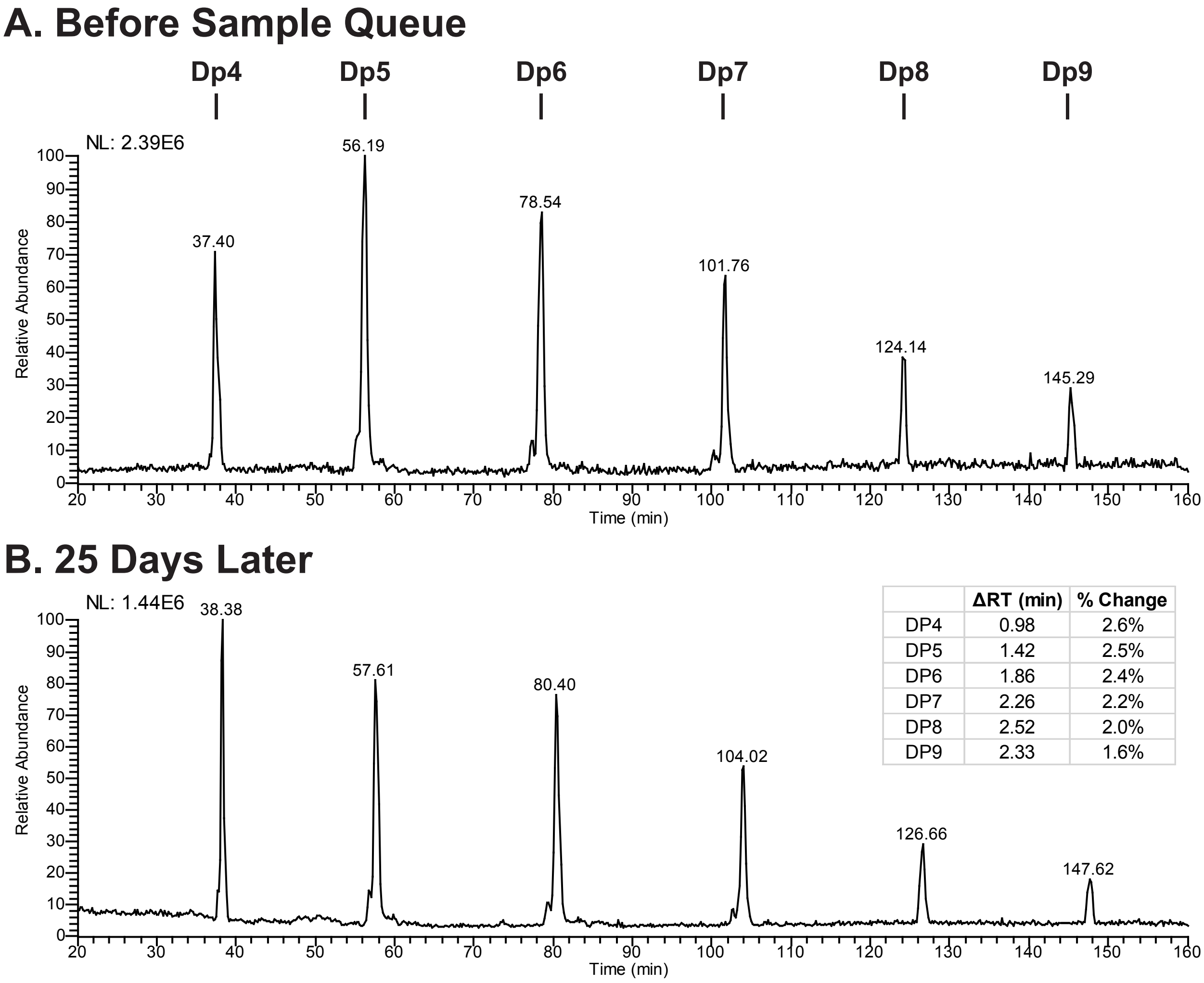

	ΔRT (min)	% Change
DP4	0.98	2.6%
DP5	1.42	2.5%
DP6	1.86	2.4%
DP7	2.26	2.2%
DP8	2.52	2.0%
DP9	2.33	1.6%

Supplement 2. Reproducibility of standard performance. (**A**) Reduced and ^{13}C-permethylated Dextran (isomaltooligosaccharide series, DP4 to 9) was separated using a PepMap Acclaim C18 column in 1 mM lithium acetate-containing mobile phases (45-70% B over 150 mins). Extracted ion chromatogram for each predicted mass is shown (within 10 ppm). (**B**) Rerun of Dextran after 25 days of continuously running samples.

Supplement 3 - Limit of Detection

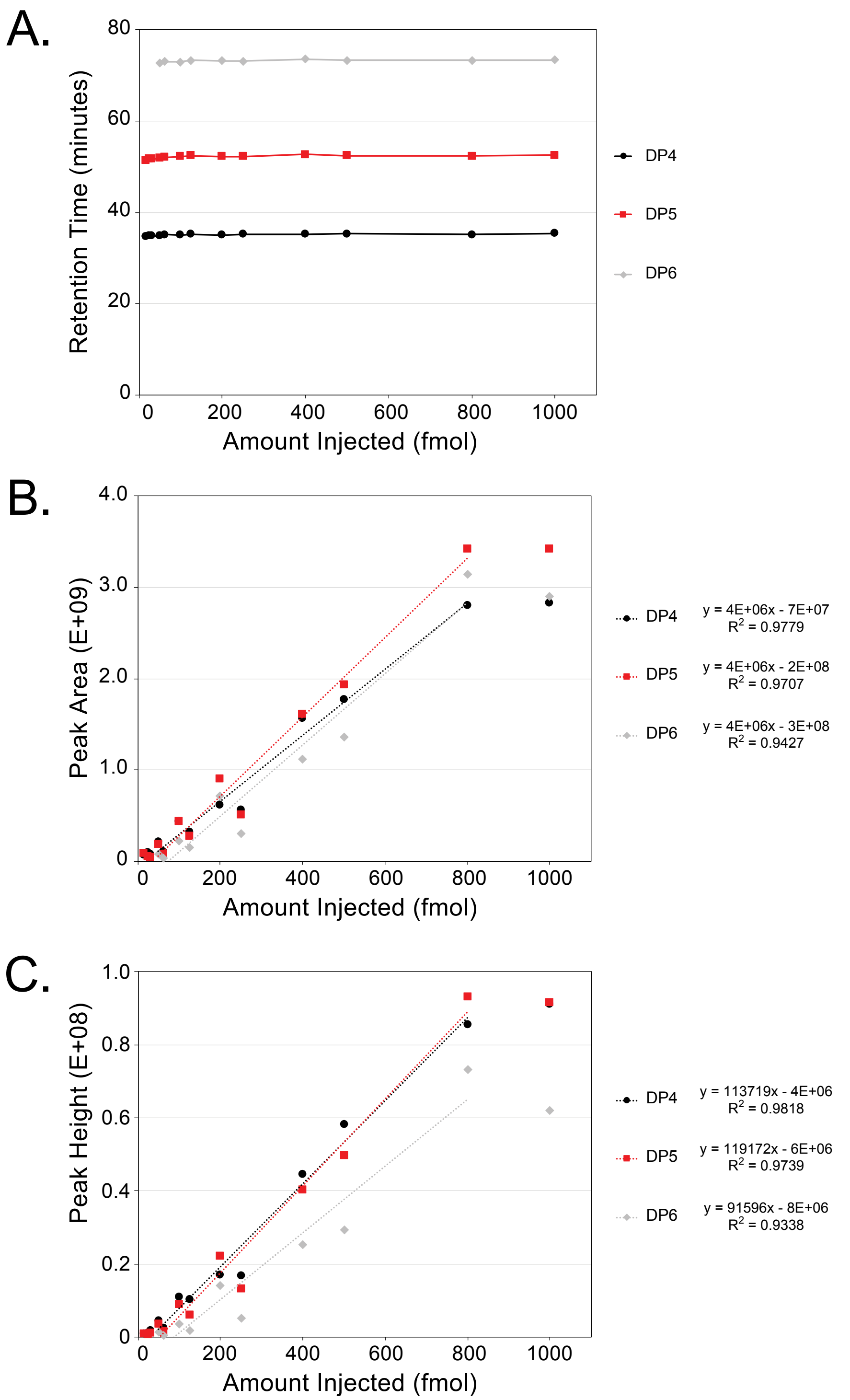

Supplement 3. Limit of Detection. Reduced and ^{13}C-permethylated isomalto analytical standards DP4-6 were analyzed by LC-NSI-MS at different amounts ranging from 15.625 to 1000 femtomoles injected and EICs were evaluated based on (**A**) Peak apex retention time, (**B**) Peak Area, and (**C**) Peak Height. Regression analysis in B and C was performed over the range 50-800 fmol injected.
